# A Systematic Review of Body Fluids Biomarkers Associated With Early Neurological Deterioration Following Acute Ischemic Stroke

**DOI:** 10.3389/fnagi.2022.918473

**Published:** 2022-05-30

**Authors:** Xiaotan Ji, Long Tian, Shumei Yao, Fengyue Han, Shenna Niu, Chuanqiang Qu

**Affiliations:** ^1^Department of Neurology, Shandong Provincial Hospital, Shandong University, Jinan, China; ^2^Department of Neurology, Jining No. 1 People’s Hospital, Jining, China; ^3^Department of Neurology, Shandong Provincial Hospital Affiliated to Shandong First Medical University, Jinan, China

**Keywords:** acute ischemic stroke, early neurological deterioration, biomarkers, body fluids, inflammation

## Abstract

Biomarkers are objectively measured biological properties of normal and pathological processes. Early neurological deterioration (END) refers to the deterioration of neurological function in a short time after the onset of acute ischemic stroke (AIS) and is associated with adverse outcomes. Although multiple biomarkers have been found to predict END, there are currently no suitable biomarkers to be applied in routine stroke care. According to the Preferred Reporting Items for Systematic Review standards, we present a systematic review, concentrating on body fluids biomarkers that have shown potential to be transferred into clinical practice. We also describe newly reported body fluids biomarkers that can supply different insights into the mechanism of END. In our review, 40 scientific papers were included. Depending on the various mechanisms, sources or physicochemical characteristics of body fluids biomarkers, we classified related biomarkers as inflammation, protease, coagulation, metabolism, oxidative stress, and excitatory neurotoxicity. The body fluids biomarkers whose related articles are limited or mechanisms are unknown are categorized as other biomarkers. The inflammation-related biomarkers, such as neutrophil-to-lymphocyte ratio and hypersensitive C-reactive protein, play a crucial role among the mentioned biomarkers. Considering the vast heterogeneity of stroke progression, using a single body fluids biomarker may not accurately predict the risk of stroke progression, and it is necessary to combine multiple biomarkers (panels, scores, or indices) to improve their capacity to estimate END.

## Introduction

Stroke (also known as apoplexia, cerebrovascular accident) is a worldwide disease burden due to its high morbidity, mortality, and disability rates. Especially it has been the first leading cause of mortality in China (GBD 2016 Stroke Collaborators, 2019). Stroke, including ischemic stroke and hemorrhagic stroke, is characterized by damage to brain tissue caused by the sudden rupture of the blood vessels in the brain or blockage that prevents blood from flowing to the brain. Ischemic stroke, accounting for about 87% of stroke cases, is caused by obstruction of the cerebral arteries (Writing Group Members et al., 2016). In acute ischemic stroke (AIS), a sudden drop in blood flow to the brain causes all or part of the supply of oxygen and glucose to be reduced to neurons and other brain cells. The result is to trigger multiple physiological, biochemical, and molecular mechanisms that ultimately result in massive cell death and changes in the basic neural function of the ischemic cell (Seners et al., 2015).

Early neurological deterioration (END) is the deterioration of nerve function in the hours or days following AIS and leads to adverse outcomes (Seners et al., 2015). The incidence of END ranges from 2.2 to 37.5% (Siegler et al., 2013; Seners et al., 2015), depending on the severity threshold and interval of clinical evaluation. The occurrence of END is affected by multiple factors and mechanisms, which cause disability or an increase in mortality. Collateral circulation disorder, primary site thrombus enlargement, recurrent stroke, cerebral edema, hemorrhagic transformation (HT) and epilepsy are risk factors that cause END following AIS (Siegler et al., 2013). Malignant edema and symptomatic intracranial hemorrhage are the two leading direct causes of END (Seners and Baron, 2018). The definition of END is not uniform at present, with most studies using the National Institutes of Health Stroke Score (NIHSS) definition of an increase of four points between admission and 24 h (Seners et al., 2015). It is well known that END is closely related to poor prognosis in stroke patients. Consequently, predictive factors for END are also of great importance. The ability to identify stroke patients with a high risk of END is critical for clinicians. Current methods of identifying the high-risk individuals depend to a large extent on the evaluations of clinical and neuroimaging features which exist at the time of onset, as well as the initial response to treatment.

Biomarkers refer to biochemical indicators that can mark structural or functional changes in systems, organs, tissues, cells and subcell structure, which can be used for a wide range of aims, such as risk stratification, treatment evaluation strategy, clinical trial design, and drug development (Biomarkers Definitions Working Group, 2001). Biomarkers are mostly derived from human tissues or body fluids, including physiological, biochemical, immune, cellular and molecular changes, which have biological materiality, and human physiological and pathological correlation of measurement, and are divided into diagnostic biomarkers, prognostic biomarkers, predictive biomarkers, pharmacodynamic biomarkers, safety biomarkers and detection biomarkers (Biomarkers Definitions Working Group, 2001). In patients with AIS, according to the expression of biomarkers, patients’ conditions can be assessed and intervened as early as possible to reduce the incidence of END, and additional clinical information independent of imaging and clinical characteristics can be provided. Considering the apparent values of predicting stroke progression, we summarize recent progress in the relationships between biomarkers and END following AIS. We expect to provide the current new knowledge of biomarkers for END in AIS, aiming to distinguish these high-risk stroke patients and obtain closer monitoring and aggressive treatment, improving long-term clinical prognosis. In the meantime, we look forward to providing direction for future research and could potentially lead to the development of novel therapeutic approaches.

## Methods

The present systematic review was performed according to the Preferred Reporting Items for Systematic Review and Meta-analyses (PRISMA) standards (Liberati et al., 2009). The collected studies were systematically searched and critically reviewed. A literature search of the electronic database PubMed, Science Direct Scopus, Google Scholar, and Excerpta Medica Database (EMBASE) from database inception to October 2021 was conducted. The search terms were “acute ischemic stroke,” “cerebral infarction,” “early neurological deterioration,” “biomarker” in the title and abstract. Early neurological deterioration refers to the deterioration of neurological function in a short time after the onset of acute stroke, without regard to severity threshold and interval of clinical evaluation. The references of all articles retrieved were examined and cross-referenced to further identify the related literature. The selection criteria were as follows: (1) original research articles; and (2) reviews, and mini-reviews. In addition, non-English papers and case reports/series were excluded. Preprint articles were also excluded. The literature search was conducted by two researchers (TL, YSM). Two researchers (JXT, TL) independently reviewed the papers whose titles or abstracts appeared to be relevant and selected those which analyzed biomarkers associated with END flowing AIS. The dispute over the qualifications of the two researchers was resolved through negotiation. Critical appraisal and assessment of bias were accomplished by two investigators (HFY, YSM). Data extraction was conducted by two investigators (JXT, TL) and confirmed by other investigators (HFY, NSN). Synthesis of results was completed by one researcher (JXT).

## Results

**FIGURE 1:**
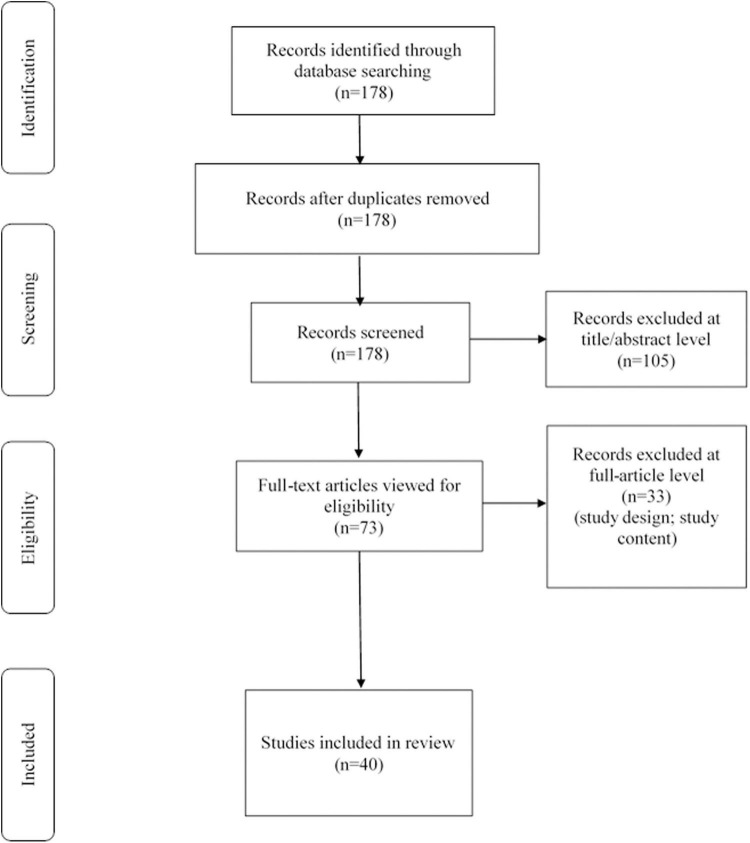
Search strategy: A methodological evaluation of each study was examined according to the PRISMA standards, including an assessment of bias.

We reviewed the titles and abstracts, as well as manually searched the reference lists. The reference lists of all included articles were checked to avoid missing literatures. A total of 178 articles were found in the search and then screened based on their abstracts. These included papers were further viewed full-text critically, considering the primary purposes of the review. This assessment left 40 related articles comprising original research articles, reviews, or mini-reviews. Figure 1 showed our search strategy. Table 1 demonstrated related studies on the body fluids biomarkers of END flowing AIS, excluding the review, mini-review, animal experiment, and the research not mentioning the indicator of sensitivity, specificity and adjusted odds ratio (aOR) and 95% confidence interval (95% CI).

**TABLE 1:** Published studies on the biomarkers of END following AIS.

Type	Biomarker	AIS subtype	Cut-off	First sample collection (after admission)	Se (%)	Sp (%)	References
inflammation	NLR	LAAS	N/A	<24 h	N/A	N/A	Nam et al., 2019
		single subcortical infarctions	N/A	<24 h	aOR = 1.24	95%CI 1.04–1.49	Nam et al., 2021
		First-ever AIS treated with intravenous thrombolysis within 4.5 h	4.43	<4.5 h	70.9	79.3	Gong et al., 2019b
		AIS treated with intravenous thrombolysis within 4.5 h	N/A	<4.5 h	1.385	95%CI 1.238–1.551	Gong et al., 2021
		AIS received intravenous thrombolysis or endovascular thrombectomy of the anterior circulation	7	<24 h	60	60	Ferro et al., 2021
	Hs-CRP	First-ever AIS	N/A	days 1, 3, 7, 14	N/A	N/A	Zang et al., 2016
		AIS with AF	N/A	<24 h	2.78	95%CI 1.067–7.240	Duan et al., 2020
		penetrating artery infarction	3.48 mg/L	day 2	73.64	82.35	Gong et al., 2019a
	IL-6	AIS received endovascular therapy	28.04 pg/mL	days 1, 2, 3, 7 <24 h	1.98	95%CI 1.05–6.69	Deng et al., 2021
protease	MMP-9	AIS within 24 h	181.7 ng/mL	<24 h	0.829	0.813	Yuan et al., 2018
	ALP	AIS with AF and/or rheumatic heart disease	N/A	<48 h	aOR = 8.96	95%CI 1.33–60.21	Liu et al., 2016
		AIS caused by intracranial atherosclerosis	N/A	<day 1	N/A	N/A	Uehara et al., 2020
	Lp-PLA 2	first-ever AIS	2.99	<48 h	1.96	95%CI 1.02–4.27	Wang et al., 2019
coagulation	P-selectin	AIS	N/A	days 1, 3, 7 and 14	N/A	N/A	Wang et al., 2013
	CLEC-2	AIS within 7 days	235.48 pg/ml	<7 days	76.8	54.2	Zhang et al., 2018
	D-dimer	AIS	N/A	<24 h	1.87	95%CI 1.38–2.54	Barber et al., 2004
		AIS	N/A	<24 h	N/A	N/A	Barber et al., 2006
		AIS within 24 h	N/A	<24 h	3.622	95%CI 1.732–7.573	Liu et al., 2020
metabolite	blood glucose	AIS treated with recanalization treatment (EVT after IV rt-PA, EVT, or IV rt-PA alone)	107.1 mg/dL	<24 h	100	53	Huang Z. X. et al., 2020
	glycemic variability	AIS with type 2 diabetes	N/A	<72 h	1.479	95%CI 1.162–1.882	Hui et al., 2018
	glycated albumin	AIS with prediabetes	N/A	<12 h	4.58	95%CI 1.64–12.81	Lee et al., 2021
	HDL-cholesterol	AIS within 24 h	N/A	<24 h	0.42	95% CI 0.19–0.94	Ryu et al., 2016
	apoB/apoA-I ratio	AIS within 24 h	N/A	<24 h	2.37	95% CI 1.02–5.53	Ryu et al., 2016
	triglyceride	AIS	N/A	<24 h	N/A	N/A	Choi et al., 2012
	Cystatin C	AIS and TIA in elderly patients without chronic kidney disease	N/A	<24 h	N/A	N/A	Kim et al., 2017
		cardiogenic cerebral embolism	1.41 mg/L	<24 h	49.1	75.7	Cong and Ma, 2021
	WBPC TMAO	AIS within 4.5 h AIS within 24 h	6.05 μMc 5.0 μmol/L	at admission <24 h	N/A 2.14	N/A 95%CI 1.19–3.82	Martin et al., 2019; Hou et al., 2020
others	Albuminuria	acute small subcortical infarcts in the lenticulostriate artery territory within 24 h	N/A	the first morning after admission	6.64	1.62–27.21	Umemura et al., 2014
		AIS	N/A	at admission	5.58	95%CI 2.24–14.82	Kanamaru et al., 2017
	cTnI	AF related AIS	N/A	<24 h	1.16	95%CI 1.00–1.34	Nam et al., 2020
	homocysteine	AIS within 48 h	11.4 μmol/L	<24 h	3.45	95%CI 1.25–9.50	Kwon et al., 2014

*Wherever appropriate, the sensitivity (Se) and specificity (Sp) have been replaced by the adjusted odds ratio (aOR) and the corresponding 95% confidence interval (CI), respectively. 1. An indicated combination of D-dimer level and platelet count. 2. Prediabetes, including impaired fasting glucose and/or impaired glucose tolerance (IGT) and/or impaired hemoglobin A1c (HbA1c), is an intermediate metabolic state between normal glucose metabolism and diabetes. Not included the review, mini-review, animal experiment, and the research not mentioned the indicator of Se, Sp, and aOR. AIS, acute ischemic stroke; TIA, transient ischemic attack; AF, atrial fibrillation; LAAS, large artery atherosclerosis stroke; EVT, endovascular treatment; IV rt–PA, intravenous recombinant tissue plasminogen activator; N/A, not applicable or not available; NLR, neutrophil-to-lymphocyte ratio; Hs-CRP, hypersensitive C-reactive protein; interleukin-6, IL-6; MMP-9, matrix metalloproteinase-9; ALP, Alkaline phosphatase; Lp-PLA 2, Lipoprotein-associated phospholipase A 2; F2-isoP, F2-isoprostanes; CLEC-2, C-Type Lectin-Like Receptor; HDL-cholesterol, high-density lipoprotein-cholesterol; apoB/apoA-I ratios, apolipoprotein B/apolipoprotein A-I ratios; Hcy, homocysteine; WBPC, whole blood purine concentration; TMAO, Trimethylamine N-oxide; cTnI, cardiac troponin I; GA, glycated albumin; h, hour.*

### Biomarkers Related to Inflammation

#### Neutrophil-to-Lymphocyte Ratio (NLR)

Previous studies have shown that neuroinflammation plays an essential role in the pathophysiologic process and progression of cerebral infarction (Shi et al., 2019; Stoll and Nieswandt, 2019; Parikh et al., 2020; Schuhmann et al., 2020). Neutrophils are the primary inflammatory cells in AIS, releasing pro-inflammatory cytokines and other cytotoxic products (proteases, reactive oxygen species) that cause secondary brain damage in the ischemic penumbra (Ceulemans et al., 2010). At the same time, neutrophils are the source of matrix metalloproteinase-9 (MMP-9), which directly induces blood-brain barrier (BBB) disruption and HT (Duan et al., 2018). On the other hand, specific lymphocyte subtypes, such as regulatory T cells (Tregs), are the primary brain-protective immunomodulators of ischemic injury after AIS. They have been shown associated with reduced infarct volume and improved neurological function (Kim et al., 2012; Liesz et al., 2013). Therefore, a single biochemical indicator is limited in reflecting the progression of AIS. NLR, platelet-to-lymphocyte ratio (PLR), and lymphocyte-to-monocyte ratio (LMR) are comprehensive parameters that can preferably reflect the immune activity of cells and has a better role in prediction. Recent studies suggest that NLR, PLR, and LMR are potential novel biomarkers of underlying inflammatory processes that may serve as better predictors of ischemic stroke (Lux et al., 2020). Especially elevated NLR is associated with an increased risk of carotid artery stenosis (Koklu et al., 2016) and intracranial atherosclerosis (Nam et al., 2018). Patients with large artery atherosclerosis stroke (LAAS) are prone to END and therefore have a poorer clinical prognosis (Lee and Lee, 2017). Nam et al. reported that NLR level was independently associated with END events in patients with LAAS. The mechanism might be involved in the degree of large artery stenosis, the number of stenotic lesions, and the stability of atherosclerotic plaque (Nam et al., 2019). The NLR level was also related to END in patients with single subcortical infarctions which were defined as an infarct in the area of penetrating arteries and its prognosis is relatively favorable (Nam et al., 2021).

Intravenous thrombolysis and endovascular treatment are the main methods in the treatment of AIS. However, the overall efficacy of these treatments is limited, with only 30 to 50% of patients achieving good long-term outcomes (Semerano et al., 2019). Possible causes include BBB dysfunction, brain injury secondary to cerebral edema, HT, and infarct enlargement (Mizuma et al., 2018; Shi et al., 2019). Previous studies showed that an elevated level of NLR may predict post-thrombolysis END in ischemic stroke patients. It also indicated that the optimal cutoff value of NLR indicating post-thrombolysis END was 4.43, with corresponding sensitivity and specificity of 70.9 and 79.3%, respectively (Gong et al., 2019b). The results of another observational study also demonstrated that NLR, PLR, and LMR are associated with post-thrombolysis END, where NLR and PLR may have the ability to predict (Gong et al., 2021). However, neither research explored related mechanisms. Ferro et al. showed that NLR level within 24 h was following the severity of cerebral edema and END in patients with anterior circulation infarction who received reperfusion therapy (including intravenous thrombolysis and intravascular thrombectomy), and cerebral edema might be an essential mechanism linking systemic inflammation to secondary brain injury (Ferro et al., 2021).

#### Hypersensitive C-Reactive Protein (Hs-CRP)

Hypersensitive C-reactive protein is a systemic marker of inflammation that is produced in large quantities by hepatocytes, and is caused by IL-1, IL-6 and TNF-α stimulation (Lakhan et al., 2009). Ischemic brain tissue releases inflammatory cytokines and chemokines, and Hs-CRP is one of the mediators of ischemic brain injury. Cytokines and inflammatory factors cause neuron necrosis, vascular endothelial cell permeability, and BBB destruction, inducing cell apoptosis (Esenwa and Elkind, 2016). The previous study has found that patients with progressive cerebral infarction had higher Hs-CRP levels than patients with non-progressive cerebral infarction in 3, 7, and 14 days after the onset of AIS (Zang et al., 2016). However, the research did not analyze the subtypes of AIS. A recent study showed that Hs-CRP levels were independently associated with END in cerebral embolism caused by atrial fibrillation (AF) (Duan et al., 2020). Although the exact mechanism behind this association is unclear, there are two possible explanations. First of all, cerebral edema after an acute cerebral infarction can trigger the body’s inflammatory response, promote inflammation in the brain, and worsen the cerebral infarction. Therefore, cerebral edema may be one of the leading causes of END. As an acute-phase reactant and systemic inflammatory indicator, Hs-CRP levels may also be elevated by cerebral edema in acute cerebral infarction (Paquissi, 2016). In addition, previous reports indicated that patients with AF are at an increased risk of thrombosis. This tendency to thrombosis is thought to be associated with abnormal changes in inflammatory biomarkers such as Hs-CRP. Therefore, elevated Hs-CRP may further increase the risk of thrombosis in patients with AF, which may lead to infarction enlargement or new thromboembolic events (Seo et al., 2012; Ryu et al., 2016).

Penetrating artery infarctions account for about 25% of all ischemic strokes, and progressive motor deficits (PMD) are common in penetrating artery infarctions during the acute stage and sometimes lead to severe disability (Li et al., 2015). Previous studies have suggested that PMD is associated with a poor prognosis of perforating artery infarction (Li et al., 2015). Gong et al. suggested that Hs-CRP was independently correlated with PMD, and its optimal cutoff value for Hs-CRP as a predictor for PMD was 3.48 mg/L, with its sensitivity and specificity were 73.64 and 82.35%, respectively (area under the curve was 0.792) (Gong et al., 2019a). Hence, it is believed that PMD is caused by biochemical abnormalities such as inflammation.

#### Interleukin-6 (IL-6)

Vascular disease is the basis of ischemic stroke, and the role of inflammatory factors in it can’t be ignored. IL-6 is a multipotent cytokine that plays an important role in host defense by modulating immune and inflammatory responses (Lambertsen et al., 2012). IL-6 is mainly produced by macrophages, T lymphocytes and B lymphocytes, and can be induced by a variety of cytokines such as viruses, endotoxins and tumor necrosis factors in tissue damage or infection. The expression of IL-6 is strictly regulated, but persistent dysregulation of IL-6 synthesis has pathological effects on chronic inflammation and autoimmunity. As an important member of the cytokine regulatory network, IL-6 plays a core role in acute severe reactions. Moreover, IL-6 can promote the expression of intercellular adhesion molecules, promote the adhesion and aggregation of inflammatory cells, accelerate the rupture of plaques, cause vulnerable plaques and thrombosis, and induce C-reactive protein (CRP) production in the liver (Lambertsen et al., 2012). Vila et al. (2000) found that the level of IL-6 increased sharply within 24 h of experimental stroke. At the same time, the patients with END after AIS have a higher expression level of IL-6 in cerebrospinal fluid and plasma than those whose clinical symptoms improved or stabilized (Vila et al., 2000). Recent research reported that AIS patients with END had an increased expression level of IL-6 compared with AIS patients without END. A previous study has suggested that IL-6 could exacerbate the damage to the brain and disrupt the proliferation of neural stem cells (Acalovschi et al., 2003). IL-6 can diagnose early inflammation more quickly. Above all, the level of IL-6 may be a reliable predictive marker and further study is required to verify the prediction.

### Biomarkers Related to Protease

#### Matrix Metalloproteinase-9 (MMP-9)

Hemorrhagic transformation is a common complication of AIS, especially in patients with cerebral embolism and intravenous thrombolysis. It can delay effective treatment and lead to a poor prognosis for AIS patients. A recent study reported that HT is an independent risk factor for END in ischemic stroke patients receiving endovascular thrombectomy (Kim et al., 2019). Therefore, early identification of patients at high risk of HT is of great significance (Alvarez-Sabin et al., 2013). Since disruption of the BBB is associated with HT, current studies have suggested that the early BBB disruption can be used as a predictor of HT. Previous studies have indicated that MMP-9 helps to break down the extracellular matrix and the basal membrane of brain blood vessels during AIS (Lucivero et al., 2007), and it can activate numerous proinflammatory cytokines and chemokines containing interleukin and tumor necrosis factor (Candelario-Jalil et al., 2009), facilitate leukocytes transport across the endothelium (Kurzepa et al., 2014). In the meanwhile, MMP-9 plays an essential role in BBB destruction, neuronal damage, and HT (Hill et al., 2012). A meta-analysis of 12 clinical studies showed that serum MMP9 levels had high sensitivity (85%) but low specificity (79%) for HT after AIS (Wang et al., 2018). Another clinical study from China also demonstrated that serum MMP-9 levels in AIS patients with spontaneous HT were significantly higher than those in patients with non-spontaneous HT and healthy controls (Yuan et al., 2018). In this research, 181.7 ng/mL was used as the cutoff value of MMP-9, with a positive predictive value of 48% and a negative predictive value of 96%. Considering MMP-9 activity is controlled by single nucleotide polymorphisms (SNPs) of the MMP-9 gene, Yi et al. (2019) pointed out that MMP-9 polymorphisms were independently associated with a higher risk of END in AIS patients with AF. In conclusion, MMP-9 is essential to HT after AIS, and further studies are needed to confirm the diverse mechanisms of MMP-9 in AIS.

#### Alkaline Phosphatase (ALP)

Alkaline phosphatase is a molecular marker of vascular calcification and plays a vital role in atherosclerosis, leading to increased vascular stiffness and decreased compliance. In addition, ALP is considered a surrogate marker for systemic inflammation, malnutrition, and metabolic syndrome, which may lead to a worsening clinical outcome in AIS patients (Kim et al., 2013). Previous research demonstrated that increased ALP levels might help identify high-risk symptomatic HT in cardioembolic stroke patients (Liu et al., 2016). The related mechanism concerned neuroinflammation and the immature and unstable vasculature (Liu et al., 2016). Uehara et al. (2020) recently found that the elevated serum ALP level on admission may predict END in symptomatic intracranial atherosclerotic cerebral infarction. The results were only statistically significant in the symptomatic intracranial artery stenosis group, not in the symptomatic intracranial artery occlusion group. These results suggest that END is due to inter-arterial re-embolization of vulnerable plaques resulting from symptomatic intracranial atherosclerosis rather than a hemodynamic mechanism due to occlusion. Increased ALP level is a surrogate marker of instability in symptomatic intracranial artery stenosis plaques (Uehara et al., 2020). Considering the multifunction of ALP, further investigations are needed to discover the potential significance of AIS progression.

#### Lipoprotein-Associated Phospholipase A2 (Lp-PLA2)

Lipoprotein-associated phospholipase A2 also known as platelet-activating factor ethylene phthalide hydrolase, is a phospholipase that facilitates the hydrolysis of oxidized phospholipids, and is secreted by macrophages T cells and mast cells in the intima of blood vessels (Huang F. et al., 2020). Lp-PLA2 is classified as secretory (PLA2-I and PLA2-II) and cytoplasmic (PLA2-III and PLA2-IV), and is a kind of vascular-specific inflammatory marker (Huang F. et al., 2020). Studies have found that Lp-PLA2 is associated with the development of cardiovascular disease. Lp-PLA2 can hydrolyze and oxidize low-density lipoprotein to release lipid proinflammatory substances such as lysophosphatidylcholine and oxidized free fatty acids which further damage vascular endothelial cells through oxidative stress mechanism, and induce the expression of adhesion factors, thus promoting the aggregation of monocytes into the lumen to form macrophages. Macrophages, in turn, phagocytose oxidize low-density lipoprotein to form foam cells, which further stimulate the proliferation of vascular endothelial cells and ultimately participate in the formation of atherosclerotic plaques (Sofogianni et al., 2018). Furthermore, Packard et al. found that Lp-PLA2 was more closely associated with cardiovascular events than CRP (Ge et al., 2016). And a Multi-Ethnic study showed that the elevated level of Lp-PLA2 increased mortality in patients with coronary heart disease and stroke, so Lp-PLA2 has a marked relation with coronary heart disease and stroke (Ge et al., 2016). Serum Lp-PLA2 level in patients with atherosclerosis and ischemic stroke was higher than that in the normal population (Li et al., 2017; Sofogianni et al., 2018). Zhou et al. analyzed 488 patients with AIS. Patients were managed according to the NIHSS score, with 3 as the median. It was found that patients above the median had a higher level of Lp-PLA2 than that below the median, which implied that Lp-PLA2 might be independently related to the severity of ischemic stroke (Zhou et al., 2018). Recent research expounded that Lp-PLA2 was a risk factor for END following AIS. In the study, 181 patients with AIS were included and 31 patients were diagnosed END within 10 days after first-ever AIS, and the detection results showed that the probability of END increases with the increase of Lp-PLA2 level (Wang et al., 2019). The study suggested that Lp-PLA2 may as an independent predictor for END following AIS.

### Biomarkers Related to Coagulation

#### P-Selectin and C-Type Lectin-Like Receptor 2 (CLEC-2)

Thrombosis is caused by abnormal adhesion and accumulation of platelets onto blood vessel walls, resulting in interruption of blood flow to the brain. Platelet activation plays an essential role in the occurrence and development of AIS. P-selectin and CLEC-2 are both markers of platelet activation. The level of P-selectin reflects the degree of platelet activation and platelet function status, and mediates the adhesion of platelets, neutrophils and endothelial cells, which increases reperfusion injury after AIS (Wang et al., 2013). It has two types: surface membrane type and soluble type. Wang et al. found that soluble P-selectin level was high in patients with progressive AIS, especially in the progressive aortic atherosclerosis group. Meanwhile, this result was closely correlated with the onset time of progressive AIS (Wang et al., 2013). CLEC-2 is a C-Type Lectin-Like Receptor that is highly expressed on platelets, and is also closely related to platelet activation (Wu et al., 2019). Recent results suggested that elevated plasma CLEC-2 levels were significantly associated with progression in patients with AIS, and the optimal cutoff point for predicting stroke progression was 235.48 pg/mL with a specificity of 54.2%, the sensitivity of 76.8%, respectively (Zhang et al., 2018). At the same time, the author proposed several probable hypotheses to interpret the relationship. Firstly, plasma CLEC-2 takes part in continuous arterial thrombosis and atherosclerotic development, which are predictors of progression and poor outcomes of AIS. Secondly, CLEC-2 has been shown to facilitate thrombo-inflammation which may be important pathogenesis for infarct growth and neurological deterioration (Nieswandt et al., 2011).

#### D-Dimer

D-dimer is one of the degradation products of fibrin in circulating blood and can represent total fibrin concentration and thus serve as a biomarker for intravascular thrombosis (Zhang et al., 2021). Previous study found that D-dimer levels were significantly higher in patients with progressive AIS than in patients without progressive stroke (Barber et al., 2004). Barber et al. (2006) confirmed that D-dimer was an independent predictor of AIS progression using three different measurement methods (Barber et al., 2006). Furthermore, A recent meta-analysis demonstrated that high D-dimer levels on admission significantly increased the risk of recurrence on the 5-day diffusion-weighted imaging (DWI) in AIS patients (Yuan et al., 2021). As previous research noted, elevated D-dimer levels may reflect the ongoing or potential thrombosis and hypercoagulability (Haapaniemi and Tatlisumak, 2009). In addition, D-dimer may stimulate inflammatory processes. There have had an evidence that D-dimer itself can stimulate the synthesis and release of pro-inflammatory cytokines in monocytes (Kang et al., 2009), which may provide another mechanism in the progression of AIS. What is interesting, recent clinical research from China combined two biological predictors of D-dimer and platelet count, showing that patients with elevated D-dimer level and abnormal platelet count at the same time are at greater risk for END (Liu et al., 2020).

### Biomarkers Related to Metabolism

#### Blood Glucose

Previous research found patients with END had higher levels of blood glucose (Simonsen et al., 2016). In a retrospective study, 213 patients were included in the analysis, indicating that blood glucose level at admission may be an important predictor of END in female AIS patients, but not in males. In the meanwhile, the study pointed out the cutoff value of admission blood glucose level is 107.1 mg/dL (Huang Z. X. et al., 2020). Another indicator is blood sugar variability taking into account the continuous fluctuation in blood sugar, which is another vital part of dysglycemia (Gonzalez-Moreno et al., 2014). In diabetic patients with AIS, initial glycemic variability has been shown related to END (Hui et al., 2018). The glycemic variability assessment was obtained by calculating the standard deviation and the average amplitude of glycemic excursions from the glucose monitoring within the initial 3 hospital days (Hui et al., 2018). Recent research analyzing 215 acute ischemic stroke patients with prediabetes demonstrates that pre-stroke glycemic variability was associated with END occurrence (Lee et al., 2021). In this research, the glycemic variability was defined by glycated albumin (≥16.0%), which showed glycemic fluctuation prior to 4 weeks of stroke onset (Freitas et al., 2017). The authors considered that oxidative stress and insulin resistance played a crucial role in the association. Rapid blood sugar variability created more oxidative stress and reactive oxygen species than chronic hyperglycemia, which progressed to microvascular and macrovascular injury (Lee et al., 2021).

#### Glycated Albumin (GA)

Glycated albumin is the product of the non-enzymatic glycated reaction between glucose and serum albumin, which can reflect the blood glucose fluctuation level during 14–21 days. Glycated albumin is not only an indicator for screening and diagnosing of prediabetes and diabetes mellitus, but also a good predictor of diabetes mellitus complicated with macrovascular disease (Hsu et al., 2015). As an important glycosylated product, GA has a close correlation with diabetes, nephropathy, retinopathy, coronary heart disease and atherosclerosis (Li et al., 2013). Moreover, GA is closely related to the assessment of prognosis and recurrence risk of atherosclerotic cardiovascular disease and ischemic stroke, and has important clinical significance for monitoring the occurrence of cardiovascular and cerebrovascular adverse events. For example, clinical research reported that serum GA was associated with subclinical atherosclerosis when the concentration of GA was greater than 15.5%, and closely associated with carotid intima-media thickness and high sensitivity of C-reactive protein in non-diabetic residents, which hinted that GA might as assessment factor of carotid atherosclerosis (Li et al., 2013). Lee et al. detected the GA level in 215 patients with acute ischemic stroke with prediabetes, and found that the END occurrence rate was higher in the high level of GA group than that in the low GA group (Lee et al., 2021). In conclusion, glycated albumin may be an important risk factor for atherosclerosis, coronary heart disease, ischemic stroke and END following AIS.

#### Blood Lipid

Elevated total cholesterol and low-density lipoprotein (LDL)-cholesterol levels are well-known risk factor for cerebrovascular disease. Like the general expectation, a previous study from South Korea, including 410 AIS patients, has pointed out that low high-density lipoprotein-cholesterol levels and high apolipoprotein B/apolipoprotein A-I ratios were independently associated with END (Ryu et al., 2016). The research also pointed out that the association between high-density lipoprotein-cholesterol and END did not differ regardless of subtypes. However, the apolipoprotein B/apolipoprotein A-I ratio was independently related to END in large-artery atherosclerotic stroke only (Ryu et al., 2016). However, as for triglyceride (TG), both hypertriglyceridemia and low TG might be risk factors for poor outcomes in patients with AIS (Choi et al., 2012). Choi et al. (2012) found that TG had a non-linear, J-shaped association with END following AIS, which further emphasized both hyper TG and hypo TG can be risk factors for adverse early outcomes in AIS.

#### Cystatin C

The similarities in the hemodynamics between cerebral and renal vascular beds have drawn attention to the association between cerebral infarction and kidney damage (Umemura et al., 2014; Kanamaru et al., 2017). Although Cystatin C is a sensitive indicator of renal function, high levels of Cystatin C are an independent and significant predictor of END in elderly AIS patients with normal renal function in a Korean clinical study (Kim et al., 2017). As for the mechanism, the authors pointed out that the elevated serum Cystatin C may reflect an imbalance in elastolytic activity. This imbalance between proteases and inhibitors affects the cardiovascular system (Sai et al., 2016). Meanwhile, a high Cystatin C concentration is associated with inflammation and endothelial dysfunction, which is involved in the pathogenesis of atherosclerosis (Balta et al., 2013). These factors might explain the relation between microcirculation disorders and impaired vasodilation, which leads to END. Another recent research from China furtherly confirmed the essential role of Cystatin C in the END. Patients with cardiogenic cerebral embolism were enrolled in the study, and the results demonstrated Cystatin C was associated with END within 3 days of cardiogenic cerebral embolism (Cong and Ma, 2021). However, this study had several limitations that excluded other types of cerebral infarction and not investigated the potential mechanism deeply.

#### Whole Blood Purine Concentration (WBPC)

Purines are nitrogen-containing compounds with a short half-life released by the breakdown of adenosine triphosphate (ATP) and consumed again during oxidative phosphorylation. A recent study observed the relationship between END and WBPC in patients with AIS. Although the results lacked statistical significance, it still suggested that early WBPC may be a simple biomarker for predicting END in AIS patients (Martin et al., 2019). Overall, WBPC was higher for the END group than the non-END group, and the optimal ROC threshold was 6.05 μM. The impossible cellular mechanism is that the ischemic core ceased ATP metabolism and then increased purine production, whereas the penumbral tissue will consume ATP without replenishment, thereby elevating WBPC until perfusion is restored or cell death occurs (Martin et al., 2019). As AIS is the blockage of the cerebral artery, and the lack of blood supplying the brain results in energy metabolism disturbance. Considering the relationship between purine and cell metabolism, the potential function of purine in cerebral infarction is complicated. However, relevant researches are still lacking and more basic or clinical study should be implemented in the future.

#### Trimethylamine N-Oxide (TMAO)

TMAO is an enterogenic microbiota-associated metabolite synthesized mainly in the liver of the host. Intestinal microbes metabolize lecithin, choline and other nutrients to produce trimethylamine (TMA) which circulates into the liver through portal veins, and is oxidized by flavin monooxygenase 3 (FMO3) or another flavin monooxygenase (FMOx) to produce TMAO (Brunt et al., 2021). Circulating levels of choline, betaine and carnitine are associated with the development of cardiovascular disease and predict the risk of adverse cardiac events. But their prognostic assessment of disease depends on serum TMAO levels (Thomas and Fernandez, 2021). TMA/TMAO is a new crossover between diet, gut microbiome, atherosclerosis and thrombosis. Elevated plasma TMAO level has been shown to independently predict the risk of adverse cardiac events, which includes myocardial infarction, stroke and intervention with conventional cardiac risk factors and renal failure induced death (Xie et al., 2021). Research reported that high plasma TMAO levels were related to atherosclerosis (Xie et al., 2021). Clinical studies have found that patients with 3-year high plasma TMAO levels are more likely to develop atherosclerosis under the same conditions of cardiovascular risk factors (Tang et al., 2013). Wang et al. (2011) also verified that raised TMAO levels in mice by feeding cholinergic food or TMAO, and after a period of time, they found that atherosclerotic plaques appeared in the aortic root of the mice, and the area was proportional to the plasma TMAO level. At the same time, clinical data and *in vivo* studies in mice indicated that TMAO could increase platelet reactivity to agonists and shorten the clotting time of platelets, and raise the risk of thrombus formation (Zhu et al., 2016).

As a metabolite of an exogenous diet, TMAO has many direct or indirect associations with neurological diseases, and plays an important role in the development of neurological diseases. In Alzheimer’s disease, TMAO induces mitochondrial function and synaptic dysfunction, activates astrocytes to induce neuroinflammation, damages the BBB (Botchway et al., 2022). For example, the expression level of inflammatory factor CD44 was raised in primary human astrocytes treated with TMAO compared with the control group (Brunt et al., 2021). In Parkinson’s disease, TMAO can be used as an osmotic regulator to regulate the conformation of α -synuclein polypeptides (Chung et al., 2021). A recent study reported that patients with END following AIS were detected to have a higher TMAO level and the median concentration was 4.8 μmol/L (Hou et al., 2020). The research indicated that TMAO might be a predictive factor of END following AIS.

### Biomarkers Related to Oxidative Stress and Excitatory Neurotoxicity

#### F2-Isoprostanes (F2-isoP)

Oxidative stress has been shown to play an important role in the occurrence and development of ischemic brain injury (Kelly et al., 2008). F2-isoP is a product of non-cyclooxygenase free radical-induced neuronal arachidonic acid peroxidation of membrane phospholipids and lipoproteins (Morrow, 2005). Compared with other biomarkers of oxidative stress, F2-isoP was a stable, sensitive, and specific marker of oxidative stress (El Kossi and Zakhary, 2000). At the same time, it can be easily measured in serum, urine, and cerebrospinal fluid (Kelly et al., 2008). Lorenzano et al. (2018) found that increased plasma F2-isoP concentration in the hyperacute phase could independently predict the occurrence of increased infarct volume in patients with acute cerebral infarction. The result suggested the essential role of oxidative stress in promoting cerebral infarction progression, helping to understand the pathophysiological mechanism of ischemia and stroke progression. Indeed, the complicated relationship between oxidative stress and ischemic stroke should be validated in future studies.

#### MicroRNA-107 (miR-107)

Researches have shown that glutamate-induced neuron toxicity, known as excitatory neurotoxicity, could trigger ischemic neuron damage (Kostandy, 2012). Glutamate is the most common excitatory neurotransmitter in the nervous system, involving various physiological functions such as learning and memory in the brain (Kostandy, 2012). In the normal brain, glutamate transporters play an essential role in clearing the glutamate released in the synaptic cleft to maintain glutamate homeostasis and prevent neurotoxicity (Yang et al., 2014). To date, five glutamate transporter subtypes (EAAT1-EAAT5) have been identified. Of these, glutamate transporter 1 (GLT-1) is the most abundant subtype and is responsible for most glutamate clearance in the central nervous system (Yang et al., 2011; Kostandy, 2012). Down-regulation of GLT-1 expression leads to glutamate accumulation after cerebral ischemia, which in turn aggravates neuronal damage. Yang et al. (2014) found that plasma miR-107 level increased glutamate accumulation after cerebral ischemia by downregulating GLT-1 expression, which could be used as a new biomarker for monitoring excitatory toxicity in patients with ischemic stroke. Similarly, a recent bioinformatics analysis suggested that miR-107-5p is involved in stroke progression by inhibiting *furin* expression (Zhang et al., 2019). *Furin* is a gene belonging to the subtilisin-like proprotein convertase family, and is a critical mRNA function in the central nervous system (Sawada et al., 1997; Johansson, 1999), which directly leads to stroke by interfering with the formation of hypertension (Sawada et al., 1997; Johansson, 1999). These results suggest that plasma miR-107 may be a biomarker for predicting stroke progression.

#### Serum Total Bilirubin

Serum bilirubin is produced by hemoglobin released by the lysis of aging red blood cells in the body. It includes indirect bilirubin and direct bilirubin. Direct bilirubin is produced by indirect bilirubin in response to hepatocytes in the liver. Bilirubin is a powerful antioxidant. Its oxidative metabolites and biological proteins are sensitive markers of oxidative stress (Shibama et al., 2019). Studies have found that serum total bilirubin is related to the occurrence and prognosis of ischemic stroke (Thakkar et al., 2019). Physiological antioxidant and anti-inflammatory functions of serum total bilirubin can inhibit lipid oxidation and prevent plaque formation, and has protective effects on oxidative stress-mediated inflammation, atherosclerosis and ischemic cardiovascular and cerebrovascular diseases. In Fang et al’ s. study, 1,156 patients with symptomatic intracranial atherosclerotic stenosis were registered and the result showed that downregulated level of total bilirubin was detected in patients with severe and multiple atherosclerotic stenoses (Yu et al., 2021). In addition, the level of serum total bilirubin was decreased in patients with END compared with patients without END, which indicated serum total bilirubin may be a novel biomarker for the prediction of END following AIS (Sheng et al., 2021).

### Other Biomarkers

Considering the similarities in the hemodynamics between cerebral and renal vascular beds, as is mentioned above, Umemura et al. (2014) reported that after small subcortical infarcts in the lenticulostriate artery territory, proteinuria was independently associated with END. Another clinical research showed albuminuria quantified by the urinary albumin/creatinine ratio (UACR), was independently associated with END in patients with any subtype of AIS (Kanamaru et al., 2017).

As a routine diagnostic index of acute myocardial infarction, serum cardiac troponin I (cTnI) has high sensitivity and specificity (Thygesen et al., 2018). Meanwhile, cTnI can reflect AF-related cardiac structural changes (such as left atrial enlargement, endothelial dysfunction, and fibrosis) and secondary thrombosis (such as left atrial thrombosis, etc.) (Providencia et al., 2013a,b). Interestingly, elevated cTnI is also common in up to 34% of patients with acute ischemic stroke (Bugnicourt et al., 2010), but its exact cause is unknown. A recent clinical study from Korea found that elevated serum cTnI levels were associated with END in patients with AF-related stroke (Nam et al., 2020). However, the exact mechanism is still unclear, and further prospective studies are needed to verify.

Homocysteine is a sulfur-containing amino acid, which is an important intermediate product in the metabolism of methionine and cysteine. Under normal circumstances, homocysteine can be catabolized and its concentration is maintained at low levels. About 99% of homocysteine in the body is in the form of oxidized dimercapto and about 1% reduced free mercapto homocysteine. More and more studies have shown that hyperhomocysteine can cause vascular damage and lead to cardiovascular and cerebrovascular diseases (Ganguly and Alam, 2015). In 1969, the theory that homocysteine is involved in atherosclerosis was first proposed (Boushey et al., 1995). Reports suggested that homocysteine mediated vascular endothelial injury, smooth muscle cell proliferation, inflammation and oxidative stress, which were the progression of stroke and atherosclerosis, and homocysteine promoted the expression of thrombotic factors, platelet adhesion and aggregation (Zhang et al., 2020). In ischemic stroke risk factors analysis, a higher level of homocysteine was verified in the ischemic stroke group, compared with the control group (Zhang et al., 2020). Meanwhile, homocysteine plays an important role in the nervous system (Ganguly and Alam, 2015). A high level of homocysteine was an important factor to induce Alzheimer’s and Parkinson’s disease (Yin et al., 2021). Research showed that patients with a high level of serum homocysteine had a higher probability of END, which suggested that serum homocysteine could be an independent predictor for END following AIS (Kwon et al., 2014).

## Conclusion

**FIGURE 2:**
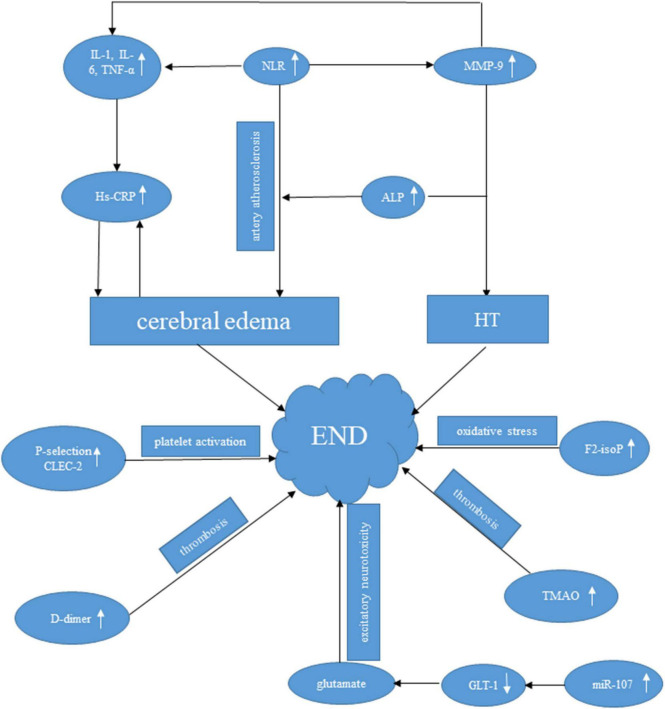
Possible mechanisms of several valuable biomarkers to END following AIS.

There are a variety of biomarkers for END (Figure 2), which provide new clues for the related mechanisms of stroke progression. Furtherly, biomarkers supply a basis for selecting clinical treatment plans, thus improving the treatment effect of END. Above all, the inflammation-related biomarkers, such as NLP, Hs-CRP, and IL-6 play a crucial role among the mentioned biomarkers. On one hand, the increased NLP indicates a high level of neutrophils, which promote the release of MMP-9 and reactive oxygen species (Ceulemans et al., 2010; Duan et al., 2018). MMP-9 then destructs BBB (Hill et al., 2012), leading to the HT occurrence. On the other hand, Hs-CRP can be triggered by cerebral edema after AIS (Paquissi, 2016) and elevated CRP may further increase the risk of thrombosis (Ryu et al., 2016). However, there is currently no biomarker used for clinical practice because of lacking high sensitivity and specificity. Further research should focus more on prospective studies and standardize the analytical methods. Considering the vast heterogeneity of stroke progression, using a single biomarker may not accurately predict the risk of stroke progression, and it is necessary to combine multiple biomarkers (panels, scores, or indices) to improve their capacity to estimate END. There is no doubt that translating biomarkers of stroke progression into clinical practice will be of great importance in improving the early identification of such patients, so that treatment regimens can be adjusted in time to improve clinical prognosis.

## Author Contributions

CQ and XJ: conceptualization. LT: methodology. FH: validation. SY and SN: formal analysis. XJ: writing original draft preparation. CQ: Reviewing and editing. All authors contributed to the drafting, critical revision of the work, and read and agreed to the published version of the manuscript.

## Conflict of Interest

The authors declare that the research was conducted in the absence of any commercial or financial relationships that could be construed as a potential conflict of interest.

## Publisher’s Note

All claims expressed in this article are solely those of the authors and do not necessarily represent those of their affiliated organizations, or those of the publisher, the editors and the reviewers. Any product that may be evaluated in this article, or claim that may be made by its manufacturer, is not guaranteed or endorsed by the publisher.

## Abbreviations

TNF-α: tumor necrosis factor α
IL-1: interleukin 1
IL-6: interleukin 6
NLR: neutrophil-to-lymphocyte ratio
Hs-CRP: hypersensitive C-reactive protein
MMP-9: matrix metalloproteinase-9
ALP: Alkaline phosphatase
F2-isoP: F2-isoprostanes
HT: Hemorrhagic transformation
END: early neurological deterioration
CLEC-2: C-Type Lectin-Like Receptor
GLT-1: glutamate transporter 1
TMAO: Trimethylamine N-oxide.

## References

[B1] Acalovschi D. Wiest T. Hartmann M. Farahmi M. Mansmann U. Auffarth G. U. (2003). Multiple levels of regulation of the interleukin-6 system in stroke. *Stroke* 34 1864–1869. 10.1161/01.STR.0000079815.38626.44 12843357

[B2] Alvarez-Sabin J. Maisterra O. Santamarina E. Kase C. S. (2013). Factors influencing haemorrhagic transformation in ischaemic stroke. *Lancet Neurol.* 12 689–705. 10.1016/S1474-4422(13)70055-3 23726850

[B3] Balta S. Demirkol S. Ay S. A. Cakar M. Sarlak H. Celik T. (2013). Serum cystatin-C levels correlate with endothelial dysfunction in patients with the metabolic syndrome. *J. Intern. Med.* 274 200–201. 10.1111/joim.12078 23600475

[B4] Barber M. Langhorne P. Rumley A. Lowe G. D. Stott D. J. (2004). Hemostatic function and progressing ischemic stroke: D-dimer predicts early clinical progression. *Stroke* 35 1421–1425. 10.1161/01.STR.0000126890.63512.41 15073383

[B5] Barber M. Langhorne P. Rumley A. Lowe G. D. Stott D. J. (2006). D-dimer predicts early clinical progression in ischemic stroke: confirmation using routine clinical assays. *Stroke* 37 1113–1115. 10.1161/01.STR.0000209240.63821.1a 16527998

[B6] Biomarkers Definitions Working Group (2001). Biomarkers and surrogate endpoints: preferred definitions and conceptual framework. *Clin. Pharmacol. Ther.* 69 89–95. 10.1067/mcp.2001.113989 11240971

[B7] Botchway B. O. Okoye F. C. Chen Y. Arthur W. E. Fang M. (2022). Alzheimer Disease: recent Updates on Apolipoprotein E and Gut Microbiome Mediation of Oxidative Stress, and Prospective Interventional Agents. *Aging Dis.* 13 87–102. 10.14336/AD.2021.0616 35111364 PMC8782546

[B8] Boushey C. J. Beresford S. A. Omenn G. S. Motulsky A. G. (1995). A quantitative assessment of plasma homocysteine as a risk factor for vascular disease. Probable benefits of increasing folic acid intakes. *JAMA* 274 1049–1057. 10.1001/jama.1995.03530130055028 7563456

[B9] Brunt V. E. Larocca T. J. Bazzoni A. E. Sapinsley Z. J. Miyamoto-Ditmon J. Gioscia-Ryan R. A. (2021). The gut microbiome-derived metabolite trimethylamine N-oxide modulates neuroinflammation and cognitive function with aging. *Geroscience* 43 377–394. 10.1007/s11357-020-00257-2 32862276 PMC8050157

[B10] Bugnicourt J. M. Rogez V. Guillaumont M. P. Rogez J. C. Canaple S. Godefroy O. (2010). Troponin levels help predict new-onset atrial fibrillation in ischaemic stroke patients: a retrospective study. *Eur. Neurol.* 63 24–28. 10.1159/000258679 19923841

[B11] Candelario-Jalil E. Yang Y. Rosenberg G. A. (2009). Diverse roles of matrix metalloproteinases and tissue inhibitors of metalloproteinases in neuroinflammation and cerebral ischemia. *Neuroscience* 158 983–994. 10.1016/j.neuroscience.2008.06.025 18621108 PMC3584171

[B12] Ceulemans A. G. Zgavc T. Kooijman R. Hachimi-Idrissi S. Sarre S. Michotte Y. (2010). The dual role of the neuroinflammatory response after ischemic stroke: modulatory effects of hypothermia. *J. Neuroinflammation* 7:74. 10.1186/1742-2094-7-74 21040547 PMC2988764

[B13] Choi K. H. Park M. S. Kim J. T. Chang J. Nam T. S. Choi S. M. (2012). Serum triglyceride level is an important predictor of early prognosis in patients with acute ischemic stroke. *J. Neurol. Sci.* 319 111–116. 10.1016/j.jns.2012.04.018 22578636

[B14] Chung S. J. Rim J. H. Ji D. Lee S. Yoo H. S. Jung J. H. (2021). Gut microbiota-derived metabolite trimethylamine N-oxide as a biomarker in early Parkinson’s disease. *Nutrition* 83:111090. 10.1016/j.nut.2020.111090 33418492

[B15] Cong L. Ma W. (2021). Early neurological deterioration in cardiogenic cerebral embolism due to nonvalvular atrial fibrillation: predisposing factors and clinical implications. *Brain Behav.* 11:e01985. 10.1002/brb3.1985 33277821 PMC7882173

[B16] Deng Q. W. Huang S. Li S. Zhai Q. Zhang Q. Wang Z. J. (2021). Inflammatory Factors as Potential Markers of Early Neurological Deterioration in Acute Ischemic Stroke Patients Receiving Endovascular Therapy - The Aisrna Study. *J. Inflamm. Res.* 14 4399–4407. 10.2147/JIR.S317147 34511974 PMC8421252

[B17] Duan Z. Guo W. Tang T. Tao L. Gong K. Zhang X. (2020). Relationship between high-sensitivity C-reactive protein and early neurological deterioration in stroke patients with and without atrial fibrillation. *Heart Lung* 49 193–197. 10.1016/j.hrtlng.2019.10.009 31699451

[B18] Duan Z. Wang H. Wang Z. Hao Y. Zi W. Yang D. (2018). Neutrophil-Lymphocyte Ratio Predicts Functional and Safety Outcomes after Endovascular Treatment for Acute Ischemic Stroke. *Cerebrovasc. Dis.* 45 221–227. 10.1159/000489401 29763889

[B19] El Kossi M. M. Zakhary M. M. (2000). Oxidative stress in the context of acute cerebrovascular stroke. *Stroke* 31 1889–1892. 10.1161/01.str.31.8.1889 10926952

[B20] Esenwa C. C. Elkind M. S. (2016). Inflammatory risk factors, biomarkers and associated therapy in ischaemic stroke. *Nat. Rev. Neurol.* 12 594–604. 10.1038/nrneurol.2016.125 27615422

[B21] Ferro D. Matias M. Neto J. Dias R. Moreira G. Petersen N. (2021). Neutrophil-to-Lymphocyte Ratio Predicts Cerebral Edema and Clinical Worsening Early After Reperfusion Therapy in Stroke. *Stroke* 52 859–867. 10.1161/Strokeaha.120.032130 33517702

[B22] Freitas P. A. C. Ehlert L. R. Camargo J. L. (2017). Glycated albumin: a potential biomarker in diabetes. *Arch. Endocrinol. Metab.* 61 296–304. 10.1590/2359-3997000000272 28699985 PMC10118799

[B23] Ganguly P. Alam S. F. (2015). Role of homocysteine in the development of cardiovascular disease. *Nutr. J.* 14:6. 10.1186/1475-2891-14-6 25577237 PMC4326479

[B24] GBD 2016 Stroke Collaborators (2019). Global, regional, and national burden of stroke, 1990-2016: a systematic analysis for the Global Burden of Disease Study 2016. *Lancet Neurol.* 18 439–458. 10.1016/S1474-4422(19)30034-1 30871944 PMC6494974

[B25] Ge P. C. Chen Z. H. Pan R. Y. Ding X. Q. Liu J. Y. Jia Q. W. (2016). Synergistic Effect of Lipoprotein-Associated Phospholipase A2 with Classical Risk Factors on Coronary Heart Disease: a Multi-Ethnic Study in China. *Cell. Physiol. Biochem.* 40 953–968. 10.1159/000453153 27941334

[B26] Gong P. Liu Y. Gong Y. Chen G. Zhang X. Wang S. (2021). The association of neutrophil to lymphocyte ratio, platelet to lymphocyte ratio, and lymphocyte to monocyte ratio with post-thrombolysis early neurological outcomes in patients with acute ischemic stroke. *J. Neuroinflammation* 18:51. 10.1186/s12974-021-02090-6 33610168 PMC7896410

[B27] Gong P. Xie Y. Jiang T. Liu Y. Wang M. Sun H. (2019b). Neutrophil-lymphocyte ratio predicts post-thrombolysis early neurological deterioration in acute ischemic stroke patients. *Brain Behav.* 9:e01426. 10.1002/brb3.1426 31566920 PMC6790313

[B28] Gong P. Liu Y. Huang T. Chen W. Jiang T. Gong Y. (2019a). The association between high-sensitivity C-reactive protein at admission and progressive motor deficits in patients with penetrating artery infarctions. *BMC Neurol.* 19:346. 10.1186/s12883-019-1538-5 31884970 PMC6935496

[B29] Gonzalez-Moreno E. I. Camara-Lemarroy C. R. Gonzalez-Gonzalez J. G. Gongora-Rivera F. (2014). Glycemic variability and acute ischemic stroke: the missing link? *Transl. Stroke Res.* 5 638–646. 10.1007/s12975-014-0365-7 25085437

[B30] Haapaniemi E. Tatlisumak T. (2009). Is D-dimer helpful in evaluating stroke patients? A systematic review. *Acta Neurol. Scand.* 119 141–150. 10.1111/j.1600-0404.2008.01081.x 18705677

[B31] Hill J. W. Poddar R. Thompson J. F. Rosenberg G. A. Yang Y. (2012). Intranuclear matrix metalloproteinases promote DNA damage and apoptosis induced by oxygen-glucose deprivation in neurons. *Neuroscience* 220 277–290. 10.1016/j.neuroscience.2012.06.019 22710064 PMC4546359

[B32] Hou L. Zhang Y. Zheng D. Shi H. Zou C. Zhang H. (2020). Increasing trimethylamine N-oxide levels as a predictor of early neurological deterioration in patients with acute ischemic stroke. *Neurol. Res.* 42 153–158. 10.1080/01616412.2019.1710416 31928326

[B33] Hsu P. Ai M. Kanda E. Yu N. C. Chen H. L. Chen H. W. (2015). A comparison of glycated albumin and glycosylated hemoglobin for the screening of diabetes mellitus in Taiwan. *Atherosclerosis* 242 327–333. 10.1016/j.atherosclerosis.2015.07.037 26247684

[B34] Huang F. Wang K. Shen J. (2020). Lipoprotein-associated phospholipase A2: the story continues. *Med. Res. Rev.* 40 79–134. 10.1002/med.21597 31140638 PMC6973114

[B35] Huang Z. X. Huang Y. Zeng J. Hao H. Petroski G. F. Lu H. (2020). Admission Glucose Levels May Increase the Risk for Early Neurological Deterioration in Females With Acute Ischemic Stroke. *Front. Neurol.* 11:548892. 10.3389/fneur.2020.548892 33250841 PMC7674944

[B36] Hui J. Zhang J. Mao X. Li Z. Li X. Wang F. (2018). The initial glycemic variability is associated with early neurological deterioration in diabetic patients with acute ischemic stroke. *Neurol. Sci.* 39 1571–1577. 10.1007/s10072-018-3463-6 29869743

[B37] Johansson B. B. (1999). Hypertension mechanisms causing stroke. *Clin. Exp. Pharmacol. Physiol.* 26 563–565. 10.1046/j.1440-1681.1999.03081.x 10405790

[B38] Kanamaru T. Suda S. Muraga K. Okubo S. Watanabe Y. Tsuruoka S. (2017). Albuminuria predicts early neurological deterioration in patients with acute ischemic stroke. *J. Neurol. Sci.* 372 417–420. 10.1016/j.jns.2016.11.007 27836107

[B39] Kang D. W. Yoo S. H. Chun S. Kwon K. Y. Kwon S. U. Koh J. Y. (2009). Inflammatory and hemostatic biomarkers associated with early recurrent ischemic lesions in acute ischemic stroke. *Stroke* 40 1653–1658. 10.1161/Strokeaha.108.539429 19265045

[B40] Kelly P. J. Morrow J. D. Ning M. Koroshetz W. Lo E. H. Terry E. (2008). Oxidative stress and matrix metalloproteinase-9 in acute ischemic stroke: the Biomarker Evaluation for Antioxidant Therapies in Stroke (Beat-Stroke) study. *Stroke* 39 100–104. 10.1161/STROKEAHA.107.488189 18063832

[B41] Kim J. M. Bae J. H. Park K. Y. Lee W. J. Byun J. S. Ahn S. W. (2019). Incidence and mechanism of early neurological deterioration after endovascular thrombectomy. *J. Neurol.* 266 609–615. 10.1007/s00415-018-09173-0 30631916

[B42] Kim J. Song T. J. Park J. H. Lee H. S. Nam C. M. Nam H. S. (2012). Different prognostic value of white blood cell subtypes in patients with acute cerebral infarction. *Atherosclerosis* 222 464–467. 10.1016/j.atherosclerosis.2012.02.042 22460048

[B43] Kim J. Song T. J. Song D. Lee H. S. Nam C. M. Nam H. S. (2013). Serum alkaline phosphatase and phosphate in cerebral atherosclerosis and functional outcomes after cerebral infarction. *Stroke* 44 3547–3549. 10.1161/STROKEAHA.113.002959 24021686

[B44] Kim T. J. Kang M. K. Jeong H. G. Kim C. K. Kim Y. Nam K. W. (2017). Cystatin C is a useful predictor of early neurological deterioration following ischaemic stroke in elderly patients with normal renal function. *Eur. Stroke J.* 2 23–30. 10.1177/2396987316677197 31008299 PMC6453184

[B45] Koklu E. Yuksel I. O. Arslan S. Bayar N. Cagirci G. Gencer E. S. (2016). Is Elevated Neutrophil-to-Lymphocyte Ratio a Predictor of Stroke in Patients with Intermediate Carotid Artery Stenosis? *J. Stroke Cerebrovasc. Dis.* 25 578–584. 10.1016/j.jstrokecerebrovasdis.2015.10.031 26706445

[B46] Kostandy B. B. (2012). The role of glutamate in neuronal ischemic injury: the role of spark in fire. *Neurol. Sci.* 33 223–237. 10.1007/s10072-011-0828-5 22044990

[B47] Kurzepa J. Kurzepa J. Golab P. Czerska S. Bielewicz J. (2014). The significance of matrix metalloproteinase (MMP)-2 and MMP-9 in the ischemic stroke. *Int. J. Neurosci.* 124 707–716. 10.3109/00207454.2013.872102 24304146

[B48] Kwon H. M. Lee Y. S. Bae H. J. Kang D. W. (2014). Homocysteine as a predictor of early neurological deterioration in acute ischemic stroke. *Stroke* 45 871–873. 10.1161/STROKEAHA.113.004099 24448992

[B49] Lakhan S. E. Kirchgessner A. Hofer M. (2009). Inflammatory mechanisms in ischemic stroke: therapeutic approaches. *J. Transl. Med.* 7:97. 10.1186/1479-5876-7-97 19919699 PMC2780998

[B50] Lambertsen K. L. Biber K. Finsen B. (2012). Inflammatory cytokines in experimental and human stroke. *J. Cereb. Blood Flow Metab.* 32 1677–1698. 10.1038/jcbfm.2012.88 22739623 PMC3434626

[B51] Lee S. H. Kim Y. Park S. Y. Kim C. Kim Y. J. Sohn J. H. (2021). Pre-Stroke Glycemic Variability Estimated by Glycated Albumin Is Associated with Early Neurological Deterioration and Poor Functional Outcome in Prediabetic Patients with Acute Ischemic Stroke. *Cerebrovasc. Dis.* 50 26–33. 10.1159/000511938 33260185

[B52] Lee S. J. Lee D. G. (2017). Distribution of atherosclerotic stenosis determining early neurologic deterioration in acute ischemic stroke. *PLoS One* 12:e0185314. 10.1371/journal.pone.0185314 28945817 PMC5612689

[B53] Li D. Wei W. Ran X. Yu J. Li H. Zhao L. (2017). Lipoprotein-associated phospholipase A2 and risks of coronary heart disease and ischemic stroke in the general population: a systematic review and meta-analysis. *Clin. Chim. Acta* 471 38–45. 10.1016/j.cca.2017.05.017 28514697

[B54] Li H. Qiu W. Hu B. Kang Z. Wu A. M. Dai Y. (2013). Ischemic volumes and early neurologic deterioration in acute brainstem infarctions with hemoglobin A1c. *Eur. Neurol.* 70 225–232. 10.1159/000351356 24008404

[B55] Li J. B. Cheng R. D. Zhou L. Wen W. S. Zhu G. Y. Tian L. (2015). What drives progressive motor deficits in patients with acute pontine infarction? *Neural Regen. Res.* 10 501–504. 10.4103/1673-5374.153703 25878603 PMC4396117

[B56] Liberati A. Altman D. G. Tetzlaff J. Mulrow C. Gotzsche P. C. Ioannidis J. P. (2009). The PRISMA statement for reporting systematic reviews and meta-analyses of studies that evaluate healthcare interventions: explanation and elaboration. *BMJ* 339:b2700. 10.1136/bmj.b2700 19622552 PMC2714672

[B57] Liesz A. Zhou W. Na S. Y. Hammerling G. J. Garbi N. Karcher S. (2013). Boosting regulatory T cells limits neuroinflammation in permanent cortical stroke. *J. Neurosci.* 33 17350–17362. 10.1523/JNEUROSCI.4901-12.2013 24174668 PMC6618366

[B58] Liu J. Wang D. Li J. Xiong Y. Liu B. Wei C. (2016). Increased Serum Alkaline Phosphatase as a Predictor of Symptomatic Hemorrhagic Transformation in Ischemic Stroke Patients with Atrial Fibrillation and/or Rheumatic Heart Disease. *J. Stroke Cerebrovasc. Dis.* 25 2448–2452. 10.1016/j.jstrokecerebrovasdis.2016.06.017 27425768

[B59] Liu Y. Li F. Sun H. Sun Y. Sun H. Zhai Y. (2020). Combined prognostic significance of D-dimer level and platelet count in acute ischemic stroke. *Thromb. Res.* 194 142–149. 10.1016/j.thromres.2020.05.021 32788106

[B60] Lorenzano S. Rost N. S. Khan M. Li H. Lima F. O. Maas M. B. (2018). Oxidative Stress Biomarkers of Brain Damage: hyperacute Plasma F2-Isoprostane Predicts Infarct Growth in Stroke. *Stroke* 49 630–637. 10.1161/STROKEAHA.117.018440 29371434 PMC5828992

[B61] Lucivero V. Prontera M. Mezzapesa D. M. Petruzzellis M. Sancilio M. Tinelli A. (2007). Different roles of matrix metalloproteinases-2 and -9 after human ischaemic stroke. *Neurol. Sci.* 28 165–170. 10.1007/s10072-007-0814-0 17690845

[B62] Lux D. Alakbarzade V. Bridge L. Clark C. N. Clarke B. Zhang L. (2020). The association of neutrophil-lymphocyte ratio and lymphocyte-monocyte ratio with 3-month clinical outcome after mechanical thrombectomy following stroke. *J. Neuroinflammation* 17:60. 10.1186/s12974-020-01739-y 32070366 PMC7026966

[B63] Martin A. J. Dale N. Imray C. H. E. Roffe C. Smith C. J. Tian F. (2019). The association between early neurological deterioration and whole blood purine concentration during acute stroke. *Biomark. Res.* 7:7. 10.1186/s40364-019-0158-y 30988953 PMC6448300

[B64] Mizuma A. You J. S. Yenari M. A. (2018). Targeting Reperfusion Injury in the Age of Mechanical Thrombectomy. *Stroke* 49 1796–1802. 10.1161/STROKEAHA.117.017286 29760275 PMC6019565

[B65] Morrow J. D. (2005). Quantification of isoprostanes as indices of oxidant stress and the risk of atherosclerosis in humans. *Arterioscler. Thromb. Vasc. Biol.* 25 279–286. 10.1161/01.ATV.0000152605.64964.c0 15591226

[B66] Nam K. W. Kim C. K. Yu S. Chung J. W. Bang O. Y. Kim G. M. (2020). Elevated troponin levels are associated with early neurological worsening in ischemic stroke with atrial fibrillation. *Sci. Rep.* 10:12626. 10.1038/s41598-020-69303-5 32724110 PMC7387448

[B67] Nam K. W. Kim T. J. Lee J. S. Park S. H. Jeong H. B. Yoon B. W. (2019). Neutrophil-to-lymphocyte ratio predicts early worsening in stroke due to large vessel disease. *PLoS One* 14:e0221597. 10.1371/journal.pone.0221597 31449547 PMC6709913

[B68] Nam K. W. Kwon H. M. Lee Y. S. (2021). Different Predictive Factors for Early Neurological Deterioration Based on the Location of Single Subcortical Infarction: early Prognosis in Single Subcortical Infarction. *Stroke* 52 3191–3198. 10.1161/STROKEAHA.120.032966 34176312

[B69] Nam K. W. Kwon H. M. Jeong H. Y. Park J. H. Kim S. H. Jeong S. M. (2018). High neutrophil to lymphocyte ratios predict intracranial atherosclerosis in a healthy population. *Atherosclerosis* 269 117–121. 10.1016/j.atherosclerosis.2017.12.035 29353226

[B70] Nieswandt B. Pleines I. Bender M. (2011). Platelet adhesion and activation mechanisms in arterial thrombosis and ischaemic stroke. *J. Thromb. Haemost.* 9 92–104. 10.1111/j.1538-7836.2011.04361.x 21781245

[B71] Paquissi F. C. (2016). The Predictive Role of Inflammatory Biomarkers in Atrial Fibrillation as Seen through Neutrophil-Lymphocyte Ratio Mirror. *J. Biomark.* 2016:8160393. 10.1155/2016/8160393 27446629 PMC4947500

[B72] Parikh N. S. Merkler A. E. Iadecola C. (2020). Inflammation, Autoimmunity, Infection, and Stroke: epidemiology and Lessons From Therapeutic Intervention. *Stroke* 51 711–718. 10.1161/STROKEAHA.119.024157 32078460 PMC7041866

[B73] Providencia R. Barra S. Paiva L. (2013a). Atrial fibrillation, elevated troponin, ischemic stroke and adverse outcomes: understanding the connection. *Clin. Res. Cardiol.* 102 701–711. 10.1007/s00392-013-0591-0 23794062

[B74] Providencia R. Paiva L. Faustino A. Botelho A. Trigo J. Casalta-Lopes J. (2013b). Cardiac troponin I: prothrombotic risk marker in non-valvular atrial fibrillation. *Int. J. Cardiol.* 167 877–882. 10.1016/j.ijcard.2012.01.093 22353438

[B75] Ryu W. S. Schellingerhout D. Jeong S. W. Nahrendorf M. Kim D. E. (2016). Association between Serum Lipid Profiles and Early Neurological Deterioration in Acute Ischemic Stroke. *J. Stroke Cerebrovasc. Dis.* 25 2024–2030. 10.1016/j.jstrokecerebrovasdis.2016.05.009 27256172

[B76] Sai E. Shimada K. Miyauchi K. Masaki Y. Kojima T. Miyazaki T. (2016). Increased cystatin C levels as a risk factor of cardiovascular events in patients with preserved estimated glomerular filtration rate after elective percutaneous coronary intervention with drug-eluting stents. *Heart Vessels* 31 694–701. 10.1007/s00380-015-0674-0 25863806

[B77] Sawada Y. Inoue M. Kanda T. Sakamaki T. Tanaka S. Minamino N. (1997). Co-elevation of brain natriuretic peptide and proprotein-processing endoprotease furin after myocardial infarction in rats. *FEBS Lett.* 400 177–182. 10.1016/s0014-5793(96)01385-3 9001393

[B78] Schuhmann M. K. Stoll G. Bieber M. Vogtle T. Hofmann S. Klaus V. (2020). CD84 Links T Cell and Platelet Activity in Cerebral Thrombo-Inflammation in Acute Stroke. *Circ. Res.* 127 1023–1035. 10.1161/CIRCRESAHA.120.316655 32762491 PMC7508294

[B79] Semerano A. Laredo C. Zhao Y. Rudilosso S. Renu A. Llull L. (2019). Leukocytes, Collateral Circulation, and Reperfusion in Ischemic Stroke Patients Treated With Mechanical Thrombectomy. *Stroke* 50 3456–3464. 10.1161/STROKEAHA.119.026743 31619153

[B80] Seners P. Baron J. C. (2018). Revisiting ‘progressive stroke’: incidence, predictors, pathophysiology, and management of unexplained early neurological deterioration following acute ischemic stroke. *J. Neurol.* 265 216–225. 10.1007/s00415-017-8490-3 28455666

[B81] Seners P. Turc G. Oppenheim C. Baron J. C. (2015). Incidence, causes and predictors of neurological deterioration occurring within 24 h following acute ischaemic stroke: a systematic review with pathophysiological implications. *J. Neurol. Neurosurg. Psychiatry* 86 87–94. 10.1136/jnnp-2014-308327 24970907

[B82] Seo W. K. Seok H. Y. Kim J. H. Park M. H. Yu S. W. Oh K. (2012). C-reactive protein is a predictor of early neurologic deterioration in acute ischemic stroke. *J. Stroke Cerebrovasc. Dis.* 21 181–186. 10.1016/j.jstrokecerebrovasdis.2010.06.002 22277294

[B83] Sheng X. Du H. Tang Y. (2021). Decreased Serum Total Bilirubin Level Predicts Early Neurological Deterioration in Patients with Acute Ischemic Stroke. *Neuropsychiatr. Dis. Treat.* 17 1977–1982. 10.2147/NDT.S315330 34168455 PMC8216736

[B84] Shi K. Tian D. C. Li Z. G. Ducruet A. F. Lawton M. T. Shi F. D. (2019). Global brain inflammation in stroke. *Lancet Neurol.* 18 1058–1066. 10.1016/S1474-4422(19)30078-X 31296369

[B85] Shibama S. Ugajin T. Yamaguchi T. Yokozeki H. (2019). Bilirubin oxidation derived from oxidative stress is associated with disease severity of atopic dermatitis in adults. *Clin. Exp. Dermatol.* 44 153–160. 10.1111/ced.13674 29869448

[B86] Siegler J. E. Boehme A. K. Kumar A. D. Gillette M. A. Albright K. C. Martin-Schild S. (2013). What change in the National Institutes of Health Stroke Scale should define neurologic deterioration in acute ischemic stroke? *J. Stroke Cerebrovasc. Dis.* 22 675–682. 10.1016/j.jstrokecerebrovasdis.2012.04.012 22727922 PMC5535077

[B87] Simonsen C. Z. Schmitz M. L. Madsen M. H. Mikkelsen I. K. Chandra R. V. Leslie-Mazwi T. (2016). Early neurological deterioration after thrombolysis: clinical and imaging predictors. *Int. J. Stroke* 11 776–782. 10.1177/1747493016650454 27188241

[B88] Sofogianni A. Alkagiet S. Tziomalos K. (2018). Lipoprotein-associated Phospholipase A2 and Coronary Heart Disease. *Curr. Pharm. Des.* 24 291–296. 10.2174/1381612824666180111110550 29332572

[B89] Stoll G. Nieswandt B. (2019). Thrombo-inflammation in acute ischaemic stroke - implications for treatment. *Nat. Rev. Neurol.* 15 473–481. 10.1038/s41582-019-0221-1 31263257

[B90] Tang W. H. Wang Z. Levison B. S. Koeth R. A. Britt E. B. Fu X. (2013). Intestinal microbial metabolism of phosphatidylcholine and cardiovascular risk. *N. Engl. J. Med.* 368 1575–1584. 10.1056/NEJMOA1109400 23614584 PMC3701945

[B91] Thakkar M. Edelenbos J. Dore S. (2019). Bilirubin and Ischemic Stroke: rendering the Current Paradigm to Better Understand the Protective Effects of Bilirubin. *Mol. Neurobiol.* 56 5483–5496. 10.1007/s12035-018-1440-y 30612336

[B92] Thomas M. S. Fernandez M. L. (2021). Trimethylamine N-Oxide (TMAO), Diet and Cardiovascular Disease. *Curr. Atheroscler. Rep.* 23:12. 10.1007/s11883-021-00910-x 33594574

[B93] Thygesen K. Alpert J. S. Jaffe A. S. Chaitman B. R. Bax J. J. Morrow D. A. (2018). [Fourth universal definition of myocardial infarction (2018)]. *Kardiol. Pol.* 76 1383–1415. 10.5603/KP.2018.0203 30338834

[B94] Uehara T. Yoshida K. Terasawa H. Shimizu H. Kita Y. (2020). Increased serum alkaline phosphatase and early neurological deterioration in patients with atherothrombotic brain infarction attributable to intracranial atherosclerosis. *eNeurologicalSci* 20:100253. 10.1016/j.ensci.2020.100253 32695891 PMC7364112

[B95] Umemura T. Senda J. Fukami Y. Mashita S. Kawamura T. Sakakibara T. (2014). Impact of albuminuria on early neurological deterioration and lesion volume expansion in lenticulostriate small infarcts. *Stroke* 45 587–590. 10.1161/STROKEAHA.113.003164 24302481

[B96] Vila N. Castillo J. Davalos A. Chamorro A. (2000). Proinflammatory cytokines and early neurological worsening in ischemic stroke. *Stroke* 31 2325–2329. 10.1161/01.str.31.10.2325 11022058

[B97] Wang L. Wei C. Deng L. Wang Z. Song M. Xiong Y. (2018). The Accuracy of Serum Matrix Metalloproteinase-9 for Predicting Hemorrhagic Transformation After Acute Ischemic Stroke: a Systematic Review and Meta-Analysis. *J. Stroke Cerebrovasc. Dis.* 27 1653–1665. 10.1016/j.jstrokecerebrovasdis.2018.01.023 29598905

[B98] Wang Q. Zhao W. Bai S. (2013). Association between plasma soluble P-selectin elements and progressive ischemic stroke. *Exp. Ther. Med.* 5 1427–1433. 10.3892/etm.2013.985 23737893 PMC3671870

[B99] Wang Y. Hu S. Ren L. Lei Z. Lan T. Cai J. (2019). Lp-PLA2 as a risk factor of early neurological deterioration in acute ischemic stroke with TOAST type of large arterial atherosclerosis. *Neurol. Res.* 41 1–8. 10.1080/01616412.2018.1493850 30296199

[B100] Wang Z. Klipfell E. Bennett B. J. Koeth R. Levison B. S. Dugar B. (2011). Gut flora metabolism of phosphatidylcholine promotes cardiovascular disease. *Nature* 472 57–63. 10.1038/nature09922 21475195 PMC3086762

[B101] Writing Group Members, Mozaffarian D. Benjamin E. J. Go A. S. Arnett D. K. Blaha M. J. (2016). Heart Disease and Stroke Statistics-2016 Update: a Report From the American Heart Association. *Circulation* 133 e38–e360. 10.1161/CIR.0000000000000350 26673558

[B102] Wu X. Zhang W. Li H. You S. Shi J. Zhang C. (2019). Plasma C-type lectin-like receptor 2 as a predictor of death and vascular events in patients with acute ischemic stroke. *Eur. J. Neurol.* 26 1334–1340. 10.1111/ene.13984 31081579

[B103] Xie G. Yan A. Lin P. Wang Y. Guo L. (2021). Trimethylamine N-oxide-a marker for atherosclerotic vascular disease. *Rev. Cardiovasc. Med.* 22 787–797. 10.31083/j.rcm2203085 34565077

[B104] Yang J. L. Sykora P. Wilson D. M. III Mattson M. P. Bohr V. A. (2011). The excitatory neurotransmitter glutamate stimulates DNA repair to increase neuronal resiliency. *Mech. Ageing Dev.* 132 405–411. 10.1016/j.mad.2011.06.005 21729715 PMC3367503

[B105] Yang Z. B. Zhang Z. Li T. B. Lou Z. Li S. Y. Yang H. (2014). Up-regulation of brain-enriched miR-107 promotes excitatory neurotoxicity through down-regulation of glutamate transporter-1 expression following ischaemic stroke. *Clin. Sci.* 127 679–689. 10.1042/CS20140084 24943094

[B106] Yi X. Zhou Q. Sui G. Fan D. Zhang Y. Shao M. (2019). Matrix metalloproteinase-9 gene polymorphisms are associated with ischemic stroke severity and early neurologic deterioration in patients with atrial fibrillation. *Brain Behav.* 9:e01291. 10.1002/brb3.1291 31012282 PMC6576155

[B107] Yin G. Gan Y. Jiang H. Yu T. Liu M. Zhang Y. (2021). Direct Quantification and Visualization of Homocysteine, Cysteine, and Glutathione in Alzheimer’s and Parkinson’s Disease Model Tissues. *Anal. Chem.* 93 9878–9886. 10.1021/acs.analchem.1c01945 34229430

[B108] Yu F. Zhang L. Liao D. Luo Y. Feng X. Liu Z. (2021). Serum Bilirubin Levels and Extent of Symptomatic Intracranial Atherosclerotic Stenosis in Acute Ischemic Stroke: a Cross-Sectional Study. *Front. Neurol.* 12:714098. 10.3389/fneur.2021.714098 34512527 PMC8427197

[B109] Yuan B. Yang T. Yan T. Cheng W. Bu X. (2021). Relationships Between D-Dimer Levels and Stroke Risk as Well as Adverse Clinical Outcomes After Acute Ischemic Stroke or Transient Ischemic Attack: a Systematic Review and Meta-Analysis. *Front. Neurol.* 12:670730. 10.3389/fneur.2021.670730 34163426 PMC8215146

[B110] Yuan R. Tan S. Wang D. Wu S. Cao X. Zhang S. (2018). Predictive value of plasma matrix metalloproteinase-9 concentrations for spontaneous haemorrhagic transformation in patients with acute ischaemic stroke: a cohort study in Chinese patients. *J. Clin. Neurosci.* 58 108–112. 10.1016/j.jocn.2018.09.014 30287248

[B111] Zang R. S. Zhang H. Xu Y. Zhang S. M. Liu X. Wang J. (2016). Serum C-reactive protein, fibrinogen and D-dimer in patients with progressive cerebral infarction. *Transl. Neurosci.* 7 84–88. 10.1515/tnsci-2016-0013 28123826 PMC5234512

[B112] Zhang H. Zhang Q. Liao Z. (2019). Microarray Data Analysis of Molecular Mechanism Associated with Stroke Progression. *J. Mol. Neurosci.* 67 424–433. 10.1007/s12031-018-1247-3 30610589

[B113] Zhang P. Wang C. Wu J. Zhang S. (2021). A Systematic Review of the Predictive Value of Plasma D-Dimer Levels for Predicting Stroke Outcome. *Front. Neurol.* 12:693524. 10.3389/fneur.2021.693524 34295302 PMC8289899

[B114] Zhang T. Jiang Y. Zhang S. Tie T. Cheng Y. Su X. (2020). The association between homocysteine and ischemic stroke subtypes in Chinese: a meta-analysis. *Medicine* 99:e19467. 10.1097/MD.0000000000019467 32195946 PMC7220264

[B115] Zhang X. Zhang W. Wu X. Li H. Zhang C. Huang Z. (2018). Prognostic Significance of Plasma CLEC-2 (C-Type Lectin-Like Receptor 2) in Patients With Acute Ischemic Stroke. *Stroke* 10.1161/STROKEAHA.118.022563 [Epub ahead of print]. 30580704

[B116] Zhou F. Liu Y. Shi H. Huang Q. Zhou J. (2018). Relation between lipoprotein-associated phospholipase A2 mass and incident ischemic stroke severity. *Neurol. Sci.* 39 1591–1596. 10.1007/s10072-018-3474-3 29938341

[B117] Zhu W. Gregory J. C. Org E. Buffa J. A. Gupta N. Wang Z. (2016). Gut Microbial Metabolite TMAO Enhances Platelet Hyperreactivity and Thrombosis Risk. *Cell* 165 111–124. 10.1016/j.cell.2016.02.011 26972052 PMC4862743

